# Relationship between Body Mass Index Reference and All-Cause Mortality: Evidence from a Large Cohort of Thai Adults

**DOI:** 10.1155/2014/708606

**Published:** 2014-11-17

**Authors:** Vasoontara Yiengprugsawan, Cathy Banwell, Jiaying Zhao, Sam-ang Seubsman, Adrian C. Sleigh

**Affiliations:** ^1^National Centre for Epidemiology and Population Health, Research School of Population Health, College of Medicine, Biology and Environment, The Australian National University, Building 62, Mills Road, Acton, Canberra, ACT 2601, Australia; ^2^School of Human Ecology, Sukhothai Thammathirat Open University, Nonthaburi 11120, Thailand

## Abstract

We investigate variation in body mass index (BMI) reference and 5-year all-cause mortality using data from 87151 adult Open University students nationwide. Analyses focused on BMI reference bands: “normal” (≥18.5 to <23), “lower normal” (≥18.5 to <20.75), “upper normal” (≥20.75 to <23), and “narrow Western normal” (≥23 to <25). We report hazard ratios (HR) and 95% Confidence Intervals adjusting for covariates. Compared to lower normal, adults aged 35–65 years who were obese (BMI ≥ 30) were twice as likely to die during the follow-up (HR 2.37; 1.01–5.70). For the same group, when using narrow Western normal as the reference, the results were similar (HR 3.02; 1.26–7.22). However, different combinations of BMI exposure and reference band produce quite different results. Older age persons belonging to Asian overweight BMI category (≥23 to <25) were relatively protected from mortality (HR 0.57; 0.34–0.96 and HR 0.49; 0.28–0.84) when assessed using normal (≥18.5 to <23) and upper normal (≥20.75 to <23) as reference bands. Use of different “normal” reference produced varying mortality relationships in a large cohort of Thai adults. Caution is needed when interpreting BMI-mortality data.

## 1. Introduction

Body mass index (BMI) in kg/m^2^ has been commonly used in epidemiological studies as a predictor of morbidity and mortality [1, 2]. However, consideration of differing normal reference standards for BMI when relating body size and mortality has revealed that a broader reference band can lead to artefactual evidence that overweight protects against mortality [3].

Internationally, various studies have analysed all-cause mortality against normal reference BMI cut-offs, summarized by a systematic review of 97 articles with a majority of studies using a broad “normal” category of BMI 18.5–25 [4]. However, recent research on Western populations has used a narrower BMI reference band of 22.5 to <25 to represent the lowest mortality group. This information is captured in a pooled analysis of 19 prospective studies of 1.46 million white adults [5] and in a collaborative study of close to 900,000 participants from Western Europe and North America [2].

We note from previous research that Caucasians and Asians differ with regard to BMI and body composition [6, 7] and there are also differences with regard to BMI and morbidity among different Asian groups [8, 9]. Indeed, a report about a cohort of one million East and South Asians recently used a narrower BMI normal reference of 22.6 to <25.0 [10, 11]. Aware of these developments regarding BMI reference bands, we report whether variation in BMI reference bands produces a substantially different relationship with mortality, using our cohort of Thai adults and five-year mortality follow-up.

## 2. Methods and Procedures

This analysis is part of the overarching Thai Cohort Study, an ongoing epidemiological investigation of changing patterns of health risks and outcomes. Data derive from a research cohort of distance-learning adult students at Sukhothai Thammathirat Open University (STOU), who resided all over Thailand. The baseline comprehensive 20-page mailout health questionnaire was sent to all STOU students enrolled in 2005 noting that participation was voluntary and 87151 responded. The cohort participants share similar certain sociogeographic attributes with the general Thai population [12]. The cohort range from 15 to 87 years at baseline in 2005 (mean age was 29 years), slightly more than half were females, and half resided in urban areas.

The baseline questionnaire collected data on a wide range of topics including sociodemogeographic information, social and environmental context, and health behaviors and states. Weight and height were also recorded. Validity of self-reported weight and height has previously been tested among 741 STOU students and showed strong correlations between self-reported and measured weight and height (Spearman's correlation for men and women, resp., 0.95 and 0.97 for weight and 0.94 and 0.94 for height) [13].

Using self-reported weight and height, we then categorised individuals using body mass index (BMI) cut-offs based on the International Obesity Task Force categories for Asians [14]: underweight (BMI < 18.5), normal (18.5 ≥ BMI < 23), overweight at risk (23 ≥ BMI < 25), obese I (25 ≥ BMI < 30), and obese II (BMI ≥ 30). To calculate the mortality hazard ratio for a given BMI category, we used (separately) four reference BMI bands: “lower normal” (≥18.5 to <20.75), “upper normal” (≥20.75 to <23), “normal Asian” (≥18.5 to <23), “normal Western” (≥23 to <25). We stratified the cohort into two age groups (<35 years and 35–64 years) and adjusted the hazard ratio for covariates.

For mortality data linkage, all cohort members have provided their Citizen ID number which was matched with death records from the Thai Ministry of Interior and subsequently linked with causes of death from the Ministry of Public Health. Up until March 2010, there were a total of 583 deaths among the Thai Cohort Study participants.

The covariates chosen were based on our previous reports for cohort analysis of body mass index [15]. These covariates included sociodemographic variables (age in years, sex, marital status, personal household monthly income categories, paid work hours, and urban-rural residence). Also, covariates included health states, self-assessed health at baseline, and health-risk behaviours (smoking and alcohol drinking). Analyses excluded cohort members who reported chronic metabolic or cardiovascular disorders including diabetes and hypertension at baseline (12.6% of cohort members).

## 3. Results

We report BMI category prevalence overall and by two broad age groups (<35 years and 35–65 years, Table 1). We found that underweight was relatively much more common among the younger group (18.7% versus 4.5%) while overweight and obesity were much more prevalent among the older group (11.7% versus 23.7% for overweight, 9.2% versus 23.4% for obese I category, and 2.1% versus 3.9% for obese II category). We also present the 5-year crude all-cause mortality rate which was highest for upper obese category 1 among the younger group (653.8 per 100,000 persons) and for obese category II in the older group (1919.0 per 100,000 persons). We note also that for obese II category there were relatively few deaths (6 for the younger group and 18 for the older group) making mortality estimates for this extreme BMI category less precise than corresponding estimates for all other BMI categories. Cohort attributes were described by proportion overall and by body mass index categories (Table 2). Compared to females, males were much more likely to be overweight (21.7% versus 9.7%) and obese I (19.7% versus 7.8%). Also, cohort members with higher monthly income were more likely to have higher BMI (11.3% versus 16.8% versus 23.4% for overweight and 9.0% versus 14.8% versus 24.0% for obese I category).

Using hazard ratios and controlling for potential covariates, we tested four “normal” BMI reference bands (Table 3). Compared to Asian lower normal as BMI reference, cohort members aged 35–65 years who were obese (BMI ≥ 30) were twice as likely to die during the five-year follow-up (HR 2.37, 95% CI 1.01–5.70). For the same group (BMI ≥ 30; age ≥ 35 years), when using narrow Western normal as the reference, the results were similar (HR 3.02, 95% CI 1.26–7.22) and indicated that obesity was a risk for mortality. However, some different combinations of BMI exposure and reference band produce quite different results. For example, older age persons belonging to Asian overweight BMI category (≥23 to <25) were relatively protected from mortality (HR 0.57; 0.34–0.96 and HR 0.49; 0.28–0.84) when assessed using normal (≥18.5 to <23) and upper normal (≥20.75 to <23) as reference bands.

## 4. Discussion

Our findings support previous methodological research showing that BMI-mortality relationships differ substantially according to the BMI band used as normal reference [3]. In our Thai data, using an Asian lower cut-off and narrow Western normal band revealed that obese II category (BMI ≥ 30) was a predictor of mortality among older Thai adults. Reference of Asian normal and upper normal led to the conclusion that being overweight by Asian standard (23 ≥ BMI < 25) was strongly protective against mortality. Repeated analyses excluding the 204 injury deaths revealed little change in the results.

Our results were in accordance with Western studies [2, 5] and some East and South Asian studies whereby a BMI reference band of 22.5 to <25 was used as lowest mortality reference in these populations [10]. Our results were also similar to a Singapore Chinese Health Study which noted that BMI between 18.5 and 21.4 had the least mortality risk for those aged below 65 years [16]. Other large Asian BMI studies have investigated morbidity outcomes using the Asian BMI “normal” reference band (18.5 ≥ BMI < 23), but patterns of association were different to those for mortality as shown in our study [14, 17, 18]. Results presented here add to limited evidence on BMI and mortality in Asian populations [19, 20].

Our study is strengthened by the participation of a large number of Thai adults living nationwide and, in particular, by the wide array of baseline personal and health information which we used to adjust for potential confounders, including smoking status and socioeconomic status. By modest median income, geographic residence, and sex ratio our cohort represented well the Thai population. Cohort members were better educated than average for Thailand but this was advantageous for collecting accurate self-reported demographic, health, and behavioural data used in the covariate-adjusted analyses presented here.

We note one of the limitations of the study was the relatively short period of follow-up resulting in small number of deaths for analyses. It is also possible that some deaths might be due to preexisting health conditions within the 5 years of initial follow-up. Given that the cohort members provided baseline information on doctor-diagnosed chronic conditions (cardiometabolic disorders) which could be intermediate variables in the BMI-mortality causal pathway, we have excluded these cohort members from the analyses. Future longitudinal analyses will have longer follow-up periods as the cohort ages and will refine the evidence on the BMI-mortality relationship.

## 5. Conclusion

Use of different BMI bands as “normal” reference categories could lead to different conclusions when analysing body size as a determinant of all-cause mortality. Our study has demonstrated the impacts of changing the normal reference cut-offs on mortality estimates among Thai adults. Caution is needed when interpreting BMI-mortality data. There is no consensus on the optimal reference band for various populations when analysing BMI-associated morbidity and mortality and this needs to be resolved.

## Figures and Tables

**Table 1:** Body mass index and all-cause mortality, Thai Cohort Study (2005–2010).

Body mass index categories	Proportion %			5-year crude death rate per 100,000 (*n*)^*^		
	Overall	Age groups		Overall	Age groups	
		<35 years	35–65 years		<35 years	35–65 years
Underweight (<18.5)	14.7	18.7	4.5	570.0 (72)	511.2 (59)	104.0 (12)
Lower normal (≥ 18.5 to <20.75)	29.1	33.6	17.7	488.0 (122)	424.3 (88)	154.3 (32)
Upper normal (≥20.75 to <23)	25.3	24.7	26.9	714.1 (155)	505.4 (77)	485.8 (74)
Overweight (≥23 to <25)	15.1	11.7	23.7	725.9 (94)	634.7 (46)	662.3 (48)
Obese I (≥25 to <30)	13.2	9.2	23.4	850.8 (96)	653.8 (37)	653.8 (59)
Obese II (≥30)	2.6	2.1	3.9	1075.3 (24)	463.7 (6)	1919.0 (18)

^*^ *n*: actual number of deaths; total deaths = 563 for the period 2005–2010.

**Table 2:** Cohort attributes by body mass index categories, Thai Cohort Study (2005–2010).

Cohort attributes	Proportion (row %)	Body mass index categories (column %)					
		Underweight	Lower normal	Upper normal	Overweight	Obese I	Obese II
Sociodemographic							
Males	45.3	6.1	20.9	28.6	21.7	19.7	3.0
Females	54.7	21.8	35.9	22.6	9.7	7.8	2.2
Monthly income (Baht)							
≤3000 Baht	10.1	21.0	33.1	23.6	11.2	8.3	2.8
3001–7000	30.9	19.4	33.9	24.4	11.3	9.0	2.1
7001–20000	47.5	11.9	27.4	26.5	16.8	14.8	2.6
>20000	10.6	6.4	17.6	24.5	23.4	24.0	4.1
Paid work							
≤10 hours	24.3	14.3	28.8	25.1	15.5	13.6	2.7
10–40 hours	28.2	11.6	26.9	25.9	17.0	15.7	2.8
>40 hours	35.0	15.1	30.1	25.7	14.5	12.3	2.2
Residence							
Rural	48.2	14.9	30.0	25.7	14.9	12.3	2.2
Urban	52.8	14.6	28.3	24.9	15.3	14.0	2.9

Health behaviours							
Smoking							
Never	72.3	17.9	32.4	24.6	12.7	10.1	2.3
Former	17.4	6.2	19.5	27.0	22.1	21.9	3.4
Current	10.3	6.9	22.1	27.1	20.4	20.3	3.3
Alcohol drinking							
Never	26.4	20.3	33.2	23.2	11.3	9.6	2.5
Occasional	59.8	13.3	28.7	26.1	15.8	13.5	2.5
Regular	4.9	4.9	16.2	25.8	24.5	25.0	3.6
Former	9.0	13.0	26.7	25.2	16.5	15.2	3.3
Physical activity							
0 times per week	34.7	19.1	31.5	22.4	12.3	11.7	3.0
1–3 times per week	26.9	15.8	29.9	24.5	14.3	13.1	2.5
3+ times per week	38.4	10.3	26.9	28.4	17.9	14.2	2.3
Self-assessed health							
Excellent/good	70.3	13.7	29.1	26.2	15.8	12.9	2.2
Fair	25.1	16.8	29.2	23.2	13.7	13.7	3.4
Poor/very poor	4.6	19.1	29.1	22.8	11.8	13.1	4.1

**Table 3:** Association between body mass index categories and all-cause mortality, Thai Cohort Study (2005–2010).

Body mass index categories	Adjusted hazard ratio [95% Confidence Intervals] for all-cause mortality by BMI reference bands for two age groups								
	Reference (18.5 to <23)			Reference (18.5 to <20.75)		Reference (20.75 to <23)		Reference (23 to <25)	
	<35 years	35–65 years		<35 years	35–65 years	<35 years	35–65 years	<35 years	35–65 years
Underweight (<18.5)	1.16 [0.82–1.65]	1.35 [0.66–2.74]		1.15 [0.79–1.68]	1.82 [0.81–4.08]	1.18 [0.79–1.76]	1.13 [0.54–2.35]	1.06 [0.67–1.70]	2.32 [0.99–5.22]
Lower normal (≥18.5 to <20.75)	Reference			Reference		1.02 [0.73–1.43]	0.62 [0.36–1.08]	0.93 [0.62–1.39]	1.27 [0.66–2.45]
Upper normal (≥20.75 to <23)				0.98 [0.70–1.36]	1.61 [0.93–2.81]	Reference		0.90 [0.60–1.36]	**2.05** [1.19–3.53]
Overweight (≥23 to <25)	1.09 [0.75–1.58]		**0.57** [0.34–0.96]	1.08 [0.72–1.62]	0.79 [0.41–1.51]	1.11 [0.73–1.67]	**0.49** [0.28–0.84]	Reference	
Obese I (≥25 to <30.0)	1.12 [0.74–1.69]		0.93 [0.59–1.47]	1.11 [0.71–1.73]	1.28 [0.69–2.35]	1.13 [0.73–1.77]	0.79 [0.49–1.29]	1.02 [0.62–1.68]	1.63 [0.90–2.93]
Obese II (≥30)	0.63 [0.20–1.99]		1.74 [0.79–3.81]	0.63 [0.20–1.20]	**2.37** [1.01–5.70]	0.64 [0.20–2.05]	1.47 [0.66–3.28]	0.58 [0.18–1.89]	**3.02** [1.26–7.22]

^*^Hazard ratios were adjusted for baseline covariates including sex, age in years, personal monthly income, paid work hours, rural-urban residence, self-assessed health at baseline,

smoking status, alcohol drinking status, and physical activity.

^**^Bold results were statistically significant at *P* < 0.05.
